# Visceral fat area measured with computed tomography does not predict postoperative course in Crohn´s disease patients

**DOI:** 10.1371/journal.pone.0202220

**Published:** 2018-08-22

**Authors:** Stanislaus Argeny, Dietmar Tamandl, Martina Scharitzer, Anton Stift, Michael Bergmann, Stefan Riss

**Affiliations:** 1 Department of Surgery, Medical University of Vienna, Vienna, Austria; 2 Department of Biomedical Imaging and Image Guided Therapy, Medical University of Vienna, Vienna, Austria; Indiana University, UNITED STATES

## Abstract

The role of visceral fat measured by computer tomography is yet not well defined in patients with Crohn's disease. Therefore, the present study was designed to assess the impact of visceral fat area on postoperative short-term outcome and surgical characteristics. We analyzed 95 patients, who underwent intestinal resection for symptomatic Crohn's disease at an academic tertiary referral center between 2003 and 2008. Visceral fat area was measured on preoperative computed tomography scans. Postoperative morbidity was graded according to the Clavien-Dindo classification. Visceral fat area was correlated with baseline characteristics, disease phenotype and 30-day morbidity. Body mass index and age were significantly associated with a higher visceral fat area (p = 0.001). Overall 19 (20.0%) postoperative complications were observed, of whom 7 (7.4%) patients required surgical re-intervention. No significant difference was found with regard to visceral fat area between patients with an uneventful and eventful postoperative course (no complications: median visceral fat area 52.0 cm^2^ SD 59.7, complications: 41.3 cm^2^ SD 42.8; p = 0.465). In contrast to current literature, we cannot support the role of visceral fat area for predicting postoperative course in Crohn's disease. In addition, no correlation of the visceral fat area and disease behavior was detected.

## Introduction

Despite the increase use of immunosuppressive medication, patients with Crohn´s disease (CD) still have a substantial lifetime risk to undergo abdominal surgery due to disease progression [1]. Intestinal resection for CD can be complex and challenging and associated with an eventful postoperative course [2]. Thus several factors have been analyzed that could predict short-term complications in CD [3–5].

Obesity, most commonly defined as a body mass index (BMI) ≥ 30 kg/m^2^, represents a well-known cause for longer operating time and a higher rate for complications in colorectal surgery [6, 7]. However, a number of studies reported controversial results, indicating no impact of BMI on surgical outcome [8–10]. In CD, obesity has not been regarded as a common problem as most patients are suffering from weight loss because of intestinal stenosis rather leading to malnutrition than to visceral obesity. Interestingly, recent observational studies reported increasing prevalence rate of obese patients with inflammatory bowel disease [11, 12].

Although BMI represents an easily accessible marker for defining total adipose tissue accumulation, the distribution of fat, in particular the ratio between visceral and subcutaneous fat, is not demonstrated efficiently [13]. Notably, visceral obesity including metabolic syndrome, seems to play a crucial role in predicting complications. It can also lead to technical difficulties due to reduced space and more vulnerable tissue. The adipose tissue distribution and especially the “visceral fat area” (VFA) can be accurately measured and quantified by computed tomography (CT) and has been shown to correlate well with visceral fat mass [14].

Notably, to date only a paucity of studies investigated the effect of VFA in CD patients. Li et al. found a high VFA to correlate with a higher postoperative recurrence rate [15]. Another study reported that VFA was associated with a higher 30-day morbidity rate after surgery and thus to be more suitable and accurate than BMI [16].

Due to a lack of data, the present study was designed to evaluate the influence of CT-measured VFA on the postoperative course following intestinal resection for CD. Furthermore, the association of CD and intraoperative findings was investigated.

## Material and methods

We enrolled 95 patients, who underwent intestinal resection for symptomatic CD at a single academic tertiary referral center between 2003 and 2008. The study was approved by the ethics committee of the Medical University of Vienna. Written consent was waived by the ethics committee for the retrospective analysis. Patient data was anonymized prior to image analysis and readers were blinded to the clinical data.

We included only those patients with CD, who had an abdominal CT scan within 30 days prior to surgery. Patients with an MRI-enterography or no radiological work up were excluded for further analysis in order to ensure comparability of measurement. CD was confirmed by histological examination of resected specimen.

All operations were conducted or supervised by a single colorectal team specializing in the treatment of CD. The surgical technique for the laparoscopic approach has already been described elsewhere in more detail [3, 17]. Conversion was defined as extension of the planned incision.

Demographic and relevant clinical data were obtained from the institutional database and individual chart review respectively. Exposure to steroids was defined as steroid intake until the day before surgery. Azathioprin/6-mercaptopurin (AZA/6MP) treatment was registered within 2 weeks prior to surgery, whereas anti-TNF antibody therapy was documented within 1 week preoperatively.

Postoperative complications (30-day morbidity) were assessed according to the Clavien-Dindo Classification [18].

### Measurement of visceral fat area

**Fig 1:**
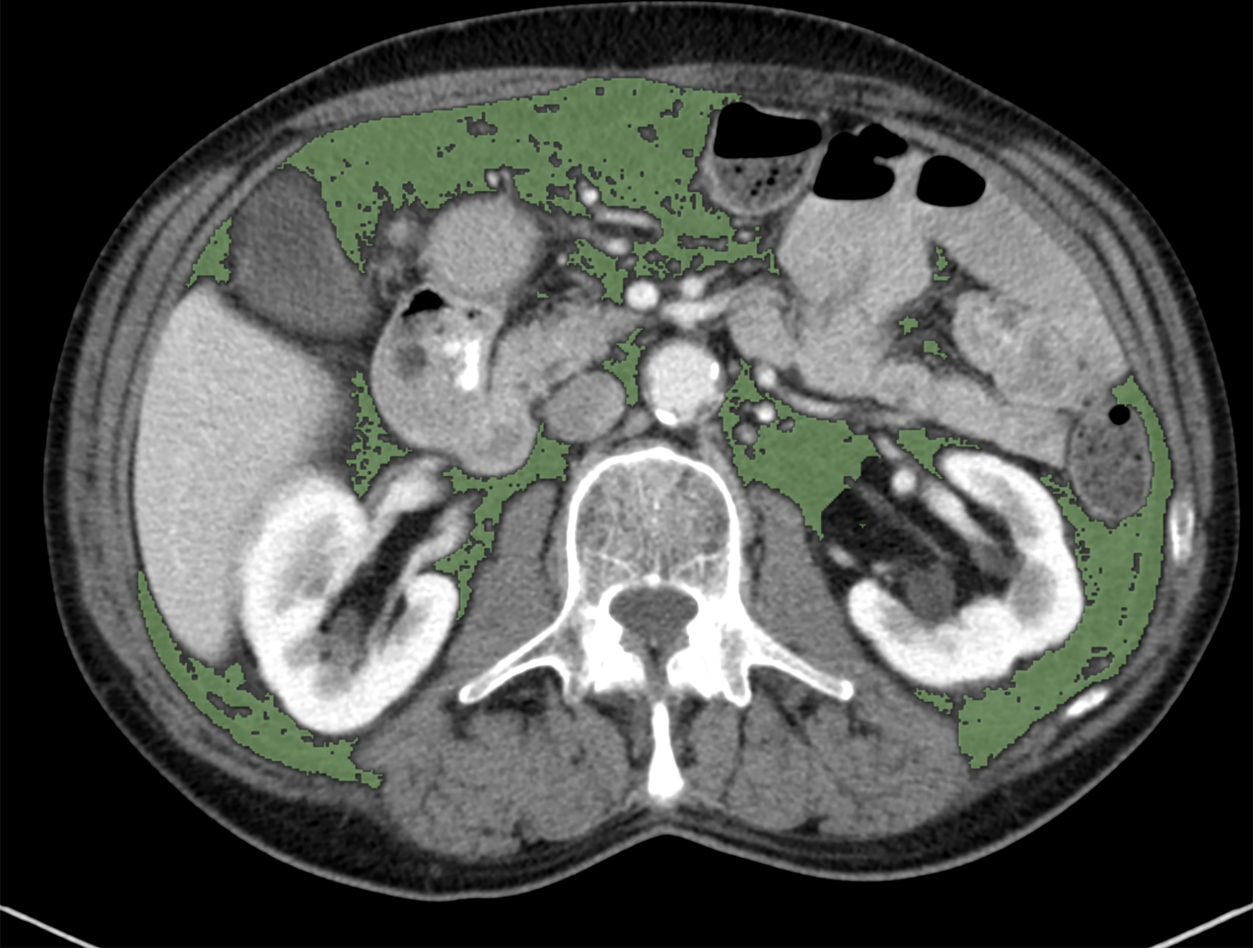
Measurement of VFA on the level of L3. From an axial CT image segmentation of visceral fat is performed in the range of -150 to -50 HU.

For the analysis of CT images, venous-phase axial images of the abdomen were exported to a workstation using OSIRIX V5.0 (Pixmeo, Sarl, Switzerland). A single slice on the level of L3, with both transverse processes visible, was selected. Semi-automated, specific tissue demarcation was performed using Hounsfield units between −150 and −50 for the delineation of visceral adipose tissue, as described previously [19]. Manual corrections were performed in case other structures outside the respective compartment were detected (Fig 1). Using this method, the VFA (cm^2^) was calculated, which has been shown to correlate well with total abdominal fat volume [20]. Furthermore the visceral fat index (VFI) was calculated (VFA/m^2^ body height) in order to correct for patients body height.

### Statistical analysis

Continuous data are shown as mean with standard deviation if normally distributed. Categorical variables are described with absolute numbers and percentages. All p-values were two-sided and p≤0.05 was considered statistically significant. All calculations were performed with SAS version 9.3 (SAS Institute Inc., Cary, NC, USA).

## Results

### Demographic data and visceral fat

Demographic characteristic of enrolled patients are listed in Table 1.

**Table 1:** Demographic characteristics of patients operated on for Crohn´s disease in correlation with visceral fat area.

Demographic data		N (%) or mean with SD	Visceral fat area^a^	p-value	Visceral fat index^b^	p-value
Sex	Female	42 (44.2)	38.0 (48.4)	0.068	14.2 (18.8)	0.239
	Male	53 (55.8)	59.3 (61.3)		19.0 (19.8)	
Age		38 (12)	49.9 (56.7)	0.001	16.9 (19.4)	0.001
BMI		21.7 (4.3)		<0.001		<0.001
Smoking	Yes	53 (55.8)	46.6 (53.7)	0.529	15.7 (18.1)	0.672
	No	42 (44.2)	54.0 (60.6)		18.4 (21.1)	
Mean CRP		3.5 (5.3)	49.9 (56.7)	0.383	16.9 (19.4)	0.323
Albumin		36.5 (6.6)		0.452		0.442
Corticosteroids	Yes	16 (16.8)	86.8 (82.3)	0.052	28.1 (26.2)	0.064
	No	79 (83.2)	42.4 (47.3)		14.6 (17.1)	
Anti TNF-antibody	Yes	0 (0.0)	0		0 (0.0)	
	No	95 (100.0)			95 (100.0)	
Azathioprine/6-mercaptopurine	Yes	19 (20.0)	38.3 (30.5)	0.150	13.0 (10.1)	0.150
	No	76 (80.0)	52.8 (61.3)		17.8 (21.0)	

Data are decribed as n (%) or mean (SD).

^a^Visceral fat area in cm^2^.

^b^Visceral fat index cm^2^/m^2^.

BMI and age were significantly correlated with a higher visceral fat (both p = 0.001). No significant differences were found with regard to sex, although male patients had a trend to higher mean VFA compared to female patients (59.3 vs. 38.0 cm^2^; p = 0.068). This difference however was lower when corrected for body height (VFI: 14.2 vs. 19.0 cm^2^/m^2^; p = 0.239).

Patients, who used corticosteroids until surgery had increased visceral fat, which did not reach a statistical significance (VFA: p = 0.052; VFI: p = 0.064). Other immunosuppressive medication and smoking history, showed a tendency towards lower visceral fat, but again were not statistically different.

### Surgical characteristics and visceral fat

In the present cohort sample we did not observe a correlation between visceral fat (VFA and VFI) and indication for surgery, surgical approach and type of resection (Table 2). Furthermore, intraoperative findings such as stenosis, fistula disease, inflammatory masses and malignant disease in the resected specimens did not correlate with elevated values of VFA nor VFI.

**Table 2:** Visceral fat area in regard to surgical characteristics in patients operated on for Crohn´s disease.

Surgical characteristics			N (%)	Visceral fat area^a^	p-value	Visceral fat index^b^	p-value
Indication	Elective		84 (88.4)	51.9 (59.0)	0.335	17.6 (20.2)	0.304
	Acute		11 (11.6)	34.3 (31.3)		11.2 (11.1)	
Surgical approach	Laparoscopic		57 (60.0)	46.8 (56.9)	0.265	15.8 (19.5)	0.442
	Laparotomy		32 (33.7)	48.5 (52.3/)		16.9 (18.8)	
	Conversion		6 (6.3)	86.4 (75.5)		26.5 (22.0)	
Type of resection	Simple (1 resection)		80 (84.2)	47.1 (53.0)	0.268	16.0 (18.2)	0.298
	Complex (>1 resection)		15 (15.8)	64.8 (73.9)		21.7 (25.0)	
Primary resection for CD	Yes		59 (62.1)	54.0 (62.1)	0.372	18.1 (21.1)	0.398
	No		36 (37.9)	43.2 (46.6)		14.7 (16.2)	
Intraoperative findings	Stenosis	Non	19 (20.0)	45.2 (44.6)	0.812	14.9 (14.6)	0.831
		1	61 (64.2)	49.4 (59.2)		17.0 (21.1)	
		>1	15 (15.8)	57.8 (62.3)		19.0 (18.4)	
	Fistula	Non	49 (51.6)	48.2 (57.8)	0.849	16.3 (20.3)	0.887
		1	31 (32.6)	48.80 (58.9)		16.7 (19.6)	
		>1	15 (15.8)	57.6 (51.3)		19.1 (17.0)	
	Inflammatory mass	Non	50 (52.6)	48.0 (54.7)	0.737	16.4 (19.3)	0.790
		Yes	45 (47.4)	52.0 (51.9)		17.4 (19.7)	
	Abscess	Non	72 (75.8)	48.2 (58.4)	0.605	16.4 (20.2)	0.668
		Yes	23 (24.2)	55.2 (51.9)		18.4 (17.1)	
Malignancy		Non	91 (95.8)	49.6 (57.0)	0.822	16.8 (19.5)	0.877
		Yes	4 (4.2)	56.2 (57.0)		18.3 (18.6)	
Perforating disease		No	41 (43.2)	47.4 (58.5)	0.716	16.3 (20.9)	0.812
		Yes	54 (56.8)	51.7 (55.7)		17.3 (18.3)	

Data are decribed as n (%) or mean (SD).

^a^Visceral fat area in cm^2^.

^b^Visceral fat index cm^2^/m^2^.

### Postoperative course and visceral fat

A total of 19 (20.0%) postoperative complications were observed and classified according to Clavien-Dindo, which are described in more detail in Table 3. Seven (7.4%) patients developed major complications requiring surgical re-intervention under general anesthesia, but no complications grade IV and V were observed.

**Table 3:** Postoperative complications of patients operated on for Crohn's disease according to the Clavien-Dindo classification.

Clavien-Dindo Classification	Complications	N (%)
Grade I	Wound Infections	4 (4.2)
	Paralytic Ileus	2 (2.1)
Grade II	Fever of Unknown Origin	3 (3.2)
	Pneumonia or Urinal Tract Infection	2 (2.1)
	Others	1 (1.1)
Grade III	Abscess	1 (1.1)
	Anastomotic Dehiscence	6 (6.3)
Grade IV	Not observed	0 (0.0)
Grade V	Not observed	0 (0.0)

Data are decribed as n (%).

Interestingly, no significant difference was found with regard to visceral fat (VFA and VFI) between patients with an uneventful and eventful postoperative course (VFA no complications: 52.0 cm^2^ SD 59.7, complications: 41.3 cm^2^ SD 42.8; p = 0.465. VFI no complications: 17.58 cm^2^/m^2^ SD 20.24, complications: 14.03 SD 15.75; p = 0.479). There was a tendency that patients with a VFI above the median VFI of the collective were more likely to be affected by grade III complications (5 vs. 2) however this was not statistical significant (p = 0.142).

Additionally, when comparing postoperative complications with BMI no significant correlation was detected too (no complications: BMI 21.8 kg/m^2^ SD 4.3, complications: 21.2 kg/m^2^ SD 4.3; p = 0.570).

Occurrence of adverse events did not differ significantly between primary and non-primary resections (n = 11 (18.6%) vs. n = 8 (22.2%); p = 0.672) S1 File.

## Discussion

In the present study we could demonstrate, that visceral fat (VFA and VFI), quantified from the preoperative CT scan, does not predict short-term outcome in patients with CD. In addition, we did not find a correlation of VFA with disease behavior, such as fistula, stenosis and inflammatory masses. These results are in contrast to previous studies [16, 21].

The impact of obesity on general surgical outcome has often been studied in literature. In CD, creeping fat with thickened mesentery is a common and typical feature, reflecting the severity of disease and subsequently challenges the surgical procedure and potentially impacts the operative outcome [22, 23].

Only a few studies investigated the effect of excessive weight on perioperative morbidity in CD. This might be attributed to the fact, that CD patients are typically not obese; however, recently, a trend to increased obesity in CD patients has been observed which might be related to better treatment options than in the past [11, 12].

Interestingly, Guardado et al. evaluated 391 patients with inflammatory bowel disease and found no association of BMI and intraoperative variables and postoperative complications [24]. In their series, 17% of all patients had a BMI ≥30 and 27% showed a BMI between 25 and 29.9.

Canedo et al. compared IBD patients with a normal BMI, who were operated on laparoscopically, with those having a BMI of above 25 [25]. Notably, of 213 patients (138 of them with CD), 19 patients were in fact obese. The authors concluded that no differences were found between both groups concerning the rate of conversion, major complications and length of hospital stay. These findings are comparable to our series, where 5 patients were defined as obese (BMI above 30 kg/m^2^) and 10 patients as overweight (BMI above 25 kg/m^2^).

In a slightly smaller cohort including 90 patients with CD, Malik et al. revealed a correlation of higher BMI with poor surgical outcome [26]. Notably, in contrast to our study only 54 patients underwent intestinal resections. Other procedures included fistulotomy, stricturoplasty and dilatation.

In general, the BMI is regarded as a reliable and comparable marker for measuring patient´s weight and thus is most frequently used in literature. Nevertheless, the distribution of fat in each individual can vary significantly according to the ethnicities, sex and also type of disease, which is not reflected by BMI solely. Bryant et al. found in a systematic review altered lean body mass and body fat in up to one third of CD patients, resulting in a misrepresentation of body composition in CD patients by BMI alone [27].

Consequently, in the recent years increased attention was paid to the distribution of fat accumulation to the mesentery, viscera and subcutaneous tissue [28]. Especially, in symptomatic CD the mesentery is typically involved and thickened due to the local inflammation process.

Erhayiem et al. found a higher mesenteric fat index (MFI), visceral to subcutaneous fat, as a marker for more aggressive disease behavior in 50 CD patients [29]. Mean age was similar to our collective and in contrast to VFA, MFI remained significantly associated with complicated CD in multivariate analysis. Notably, the measurements were conducted at lumbar vertebral level 4, which is in contrast to our study. Noteworthy, Shenet al. found in 277 patients a better representation of visceral adipose tissue at lumbar vertebral level 3, thus supporting our measurements [30]. Additionally, the authors measured a VFA of 150.1cm^2^ (SD 100.7) in complicated CD, which was higher compared to our cohort.

Ding et al. included 164 patients who underwent primary resection for symptomatic CD [16]. An overall complication rate of 38.4% was detected, which was higher compared to our results. However, VFA remained an independent risk factor for an adverse postoperative outcome in a multivariable regression analysis. Especially, in patients with visceral obesity (defined as VFA >130 cm^2^, 30.5% of the study population), postoperative adverse events occurred more frequently. Notably, only 10 (10.53%) patients in our series showed a VFA of >130 cm^2^ and mean VFA values found in patients with complications were lower in our collective than reported by Ding et al. (complication group: 41.32 cm^2^ vs. 139.88 cm^2^ and no-complication group: 52.01 cm^2^ vs. 76.12 cm^2^).

Only in one (10%) of the visceral obese patients included in our study a postoperative complication occurred, compared to 18 out of 85 (21.2%) patients in the “non visceral obese” group (p = 0.403).

Lower BMI and VFA found in our study might be partially explained by the fact that Ding et al. only included CD related primary procedures, whereas this was only the case in 59 (62.1%) of our patients, indicating a longer disease duration and consequential lower BMI [31]. Patients in our collective with primary resection had a tendency towards higher values of VFA (54.0 cm^2^ vs. 43.2 cm^2^; p = 0.372) and VFI (18.1 cm^2^/m^2^ vs. 14.7 cm^2^/m^2^; p = 0.398) compared to those with non-primary resections, but occurrence of adverse events showed no differences between the groups (p = 0.672).

Few limitations of the current study need to be addressed. Although we included a large number of patients with CD, selection bias cannot be ruled out completely. As mentioned above, we included only patients, who had a CT scan prior surgery, thus other patients were excluded from analysis. Currently, most patients in our clinic, especially those who are at a younger age, will undergo MRT enterography examination for assessing disease behavior. Therefore, not all consecutive patients were enrolled in the present analysis.

Another reason for not finding a significant correlation of VFA with an eventful course could be the low number of complications after surgery in our series as well as a lighter collective of CD patients with recurrent resective surgery, as elaborated above.

## Conclusion

This represents the second largest study investigating the influence of preoperative VFA on the postoperative course in patients undergoing intestinal resection for CD. In contrast to previous reports, we found no significant association of the VFA and 30 day morbidity. In addition, no correlation of VFA and disease behavior was detected. Consequently, we cannot support the predictive role of VFA in CD patients.

## Supporting information

S1 File Minimal data set. (XLSX)
